# The Evaluation of Artificial Intelligence Technology for the Differentiation of Fresh Human Blood Cells From Other Species' Blood in the Investigation of Crime Scenes

**DOI:** 10.7759/cureus.58496

**Published:** 2024-04-17

**Authors:** Syed Sajid Hussain Shah, Ekramy Elmorsy, Rashad Qasem Ali Othman, Asmara Syed, Syed Umar Armaghan, Syed Usama Khalid Bokhari, Mahmoud E Elmorsy, Abdulhakim Bawadekji

**Affiliations:** 1 Department of Pathology, Northern Border University, Arar, SAU; 2 Department of Pathology and Laboratory Medicine, Northern Border University, Arar, SAU; 3 Department of Research & Development – Robotic Section, Idrak AI Pvt. Ltd., Islamabad, PAK; 4 Department of Research & Development, Idrak AI Ltd., London, GBR; 5 Department of Computer Engineering, King Fahd University of Petroleum and Minerals, Dhahran, SAU; 6 Department of Biological Sciences, Northern College for Nursing, Arar, SAU

**Keywords:** blood stain, crime scene investigations, human rbc, artificial intelligence, healthcare tech contest

## Abstract

Objectives: The current study used the deep machine learning approach to differentiate human blood specimens from cow, goat, and chicken blood stains based on cell morphology.

Methods: A total of 1,955 known Giemsa-stained digitized images were acquired from the blood of humans, cows, goats, and chickens. To train the deep learning models, the well-known VGG16, Resnet18, and Resnet34 algorithms were used. Based on the image analysis, confusion matrices were generated.

Results: Findings showed that the F1 score for the chicken, cow, goat, and human classes were all equal to 1.0 for each of the three algorithms. The Matthews correlation coefficient (MCC) was 1 for chickens, cows, and humans in all three algorithms, while the MCC score was 0.989 for goats by ResNet18, and it was 0.994 for both ResNet34 and VGG16 algorithms. The three algorithms showed 100% sensitivity, specificity, and positive and negative predictive values for the human, cow, and chicken cells. For the goat cells, the data showed 100% sensitivity and negative predictive values with specificity and positive predictive values ranging from 98.5% to 99.6%.

Conclusion: These data showed the importance of deep learning as a potential tool for the differentiation of the species of origin of fresh crime scene blood stains.

## Introduction

Crime scene investigation is generally directed to biological evidence collected from a crime scene, from which blood stains are the most important [1]. Blood stains’ pattern, size, distribution, and their relationship to the surrounding items can provide important ideas that can help to predict the sequence of crime events and reconstruct the incident. In addition, biological examination of blood stains helps in the identification of the criminal [2,3].

One of the important questions in the analysis of blood stains collected from a crime scene is regarding the species of origin of the stain. Different techniques have been used to differentiate the different species of blood stains based on the morphological pattern of blood cells and other chemical techniques and physical techniques [4-6].

Red blood cells (RBCs) are the main cellular components of the human blood. It is described as rounded non-nucleated biconcave discs with approximately 7.5-8.7 μm in diameter and 1.7-2.2 μm in thickness [7]. RBCs are morphologically different from human cells in the different other species. For example, camel RBCs are oval nonnucleated corpuscles, but frog RBCs appear as elliptical nucleated cells. In addition, morphometry was used to differentiate the cells of different species. The diameter of RBCs was estimated to be 5-6 μm in cattle and horse erythrocytes and 4-5 μm in sheep erythrocytes, while goats’ RBCs showed a smaller diameter of about 2.5-3.9 μm. However, dogs showed larger RBCs in comparison to the other species (6-8 μm) [8].

The microscopic evaluation for the differentiation of human blood cells from other species is a laborious task, and it requires the services of highly qualified health professionals. The rising demand and deficiency of experts in this field create an opportunity for application of the artificial intelligence [9,10]. The application of artificial intelligence in this field of medicine could be helpful in improving the quality of results and may also reduce error possibilities attributed to human factors.

The use of a computer-aided program may be quite helpful to the health professional in improving the speed and accuracy in the microscopic evaluation of blood cells. For the development of a computer vision-based system to analyze microscopic digitized images of various human and non-human tissues, deep machine learning techniques are employed, which include different types of deep neural networks. In computational analysis of microscopic digital slides of human and non-human tissues, a convolutional neural network (CNN) is quite useful as it is most frequently applied for image analysis [11]. A CNN has an input layer, many hidden layers, and an output layer [12]. Machine learning approaches have yielded very good results when employed for the assessment of radiological images [13-15]. Computer vision-based systems have been tested for the evaluation of microscopic slides of various tumors and revealed encouraging results [16,17]. 

The computer vision-based system developed by artificial intelligence can be used for the differentiation of human blood cells from other species' blood cells during the process of investigation of various crime scenes. The objective of the present study is to apply a computer vision-based system by employing artificial intelligence for the differentiation of human blood cells from other species' blood cells based on the microscopic features and to find out the sensitivity, specificity, positive predictive value, negative predictive value, and Matthews correlation coefficient (MCC).

## Materials and methods

Ethical issues

The current study design was revised and approved ethically by the Local Committee of Bioethics at Northern Border University, Arar, Saudi Arabia (Ref. No. 441015535). A total of 360 digital images of human blood cells were acquired from the anonymized Giemsa-stained smears collected from the Bokhari Laboratory in Punjab, Pakistan, which have normocytic normochromic RBC morphology after obtaining informed consent about the study and its value. The chicken, goat, and cow samples were collected from a slaughterhouse in Punjab, Pakistan. The study was performed by using 620 digital images of RBCs of cows, 675 digitized images of RBCs of goats, and 300 images of RBCs from chickens. The technical work of AI was conducted as a joined work in the Department of Research & Development, Idrak AI Ltd., in London, United Kingdom, and Department of Research & Development - Robotic Section, Idrak AI Pvt. Ltd., Islamabad, Pakistan.

Blood smear handling

A total of 1,955 digitized images were acquired from the Giemsa-stained smear made from the blood of humans, cows, goats, and chickens. These digitized smears were evaluated by two pathologists and labeled into the respective category of the species.

The current study used deep learning algorithms to categorize RBC cell images into four groups: human, chicken, cow, and goat. To train our models, we specifically used the well-known VGG16, ResNet-18, and ResNet-34 algorithms. They are widely used in medical image analysis. The network structure of the present study was focused on the differentiation of the RBCs of the different tested species.

The ResNet network model was obtained with 18 and 34 layers and the VGG model with 16 layers as the deep neural network model of this experiment. Initially, the training set was used following data enhancement to train the four aforementioned models, and the initial learning rate was set to 0.0001. Because there have been no relevant studies or models available for transfer learning or parameter initialization before, this study selected the random initialization method. The convolution kernel was set to (3, 3), and adaptive moment estimation (Adam) was employed as an optimizer. Subsequently, a rectified linear unit (ReLU) was selected as the activation function, we applied cross-entropy to the output of the last dense layer, and the batch size was set to 32. The batch normalization module was added to each layer of the model, and weight decay (L2 regularization) was applied to avoid model overfitting. Subsequently, the epoch was set to 25, 50, 100, 150, and 200. Five models for each network structure were saved. The model with the smallest verification set loss was selected as the optimal model of the network structure. Image augmentation techniques, such as crop, zoom, flip, and rotation, were used to increase the precision of the current models using the Fast-AI library, which allowed us to modify our image data quickly and effectively.

All the functionality, experiments, and analyses were implemented using Python (NumPy 1.16 for array manipulation; opencv-python 4.1.0 and Pillow 6.0 for image operations; and scikit-learn 0.19.1 for performance quantification) and Google TensorFlow (for the implementation of the deep learning architecture).

Statistical analysis

The digitized good-quality images were processed and divided into three groups. Group 1 is the training set, which comprised 60% of the total images. Group 2 is the validation set, which contains 20%, while Group 3 is the test set, which also contains 20% of the total digitized images of the Giemsa-stained smears. TensorFlow, Keras, and Python were used for the statistical analysis.

To compare the AI findings and the standard examination, two expert pathologists participated in a comparison study. The statistical analysis compared the diagnostic results with the pathologist reports. We gave instructions to the pathologist that the examination time for each image was within 10 minutes. The pathologists were allowed to take a break for every 20 images examined.

The sensitivity, specificity, positive predictive value, negative predictive value, F1-score, and MCC were used to compare the deep learning screening performance with those of the human doctors. F1-score is the harmonic value of precision rate and recall rate. Apart from the images, the doctors were masked from all the information provided. MCC is a statistical tool used for model evaluation. Its job is to gauge or measure the difference between the predicted values and actual values. The used parameters were calculated, as shown in Table 1. A confusion matrix was used to show how many predictions are correct and incorrect per class. It helps in understanding the classes that are being confused by the model as other classes.

**Table 1:** Formulas used for the study parameters' calculations. TP: true positive, TN: true negative, FP: false positive, FN: false negative

Parameter	Formula
Sensitivity	TP/(TP + FN)
Specificity	TN/(TN + FP)
Positive predictive value	TP/TP+FP
Negative predictive value	TN/FN+TN
F1 score	2TP/2TP+FP+FN
Matthews correlation coefficient	MCC = (TP * TN - FP * FN) / sqrt((TP + FP) * (TP + FN) * (TN + FP) * (TN + FN))

## Results

Overall framework of the study

**Figure 1:**
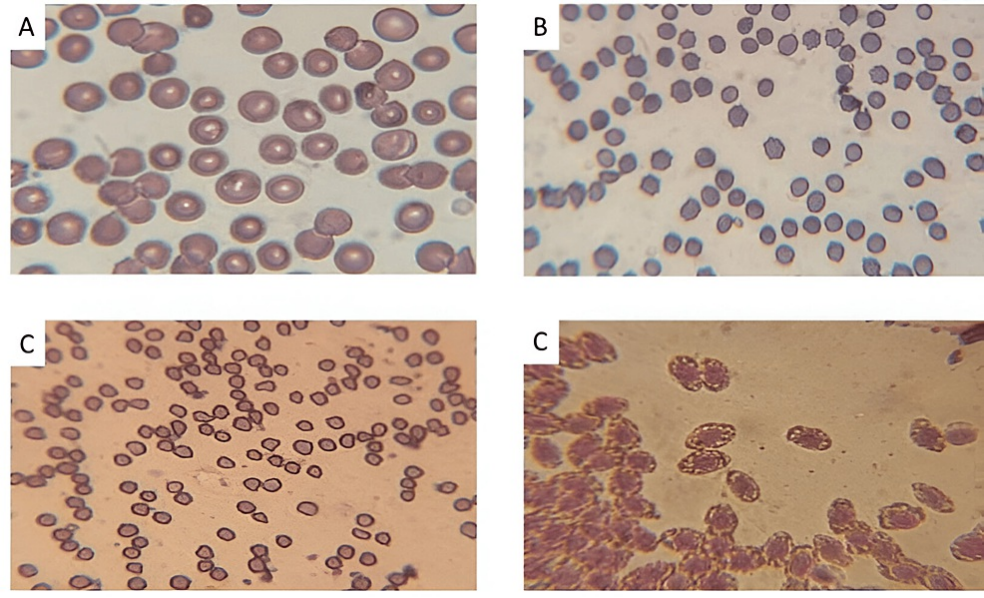
Samples of Giemsa-stained smear made from the blood of humans (A). Cow (B), goat (C), and chicken (D) samples used in the study.

The acquisition and pretraining processing of the datasets' workflow were based on a total of 1,955 images (including 360 human, 620 cow, 675 goat, and 300 chicken images) (Figure 1), which were retrieved for developing the deep learning models named VGG16, ResNet18, and ResNet34 algorithms. Finally, a total of 1,955 images were used for data augmentation to obtain 1,173 images (216 human, 372 cow, 405 goat, and 180 chicken blood film images) for training, 391 original images (72 human, 124 cow, 135 goat, and 60 chicken blood film images) for validation, and 391 original images (72 human, 124 cow, 135 goat, and 60 chicken blood film images) for testing.

**Figure 2:**
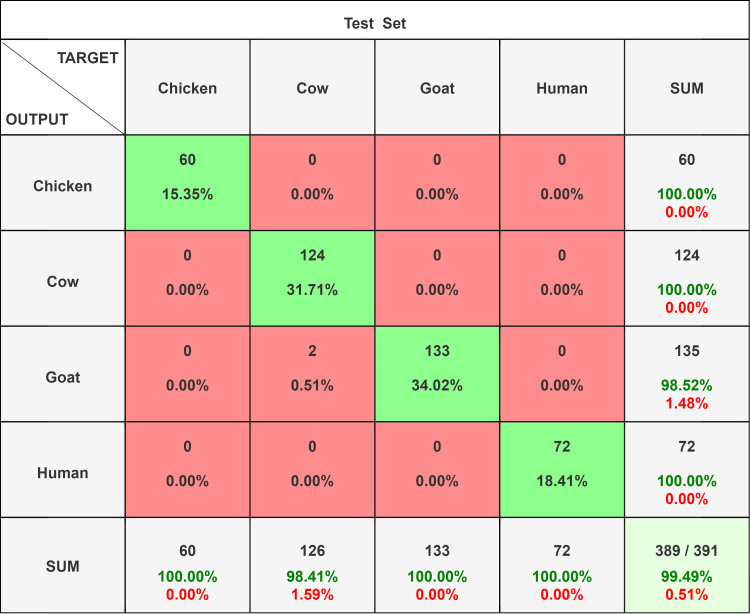
Confusion matrix comparison of different network models on test set, ResNet 18. A total of 391 (72 human, 124 cow, 135 goat, and 60 chicken blood film images) original images were used for testing

**Figure 3:**
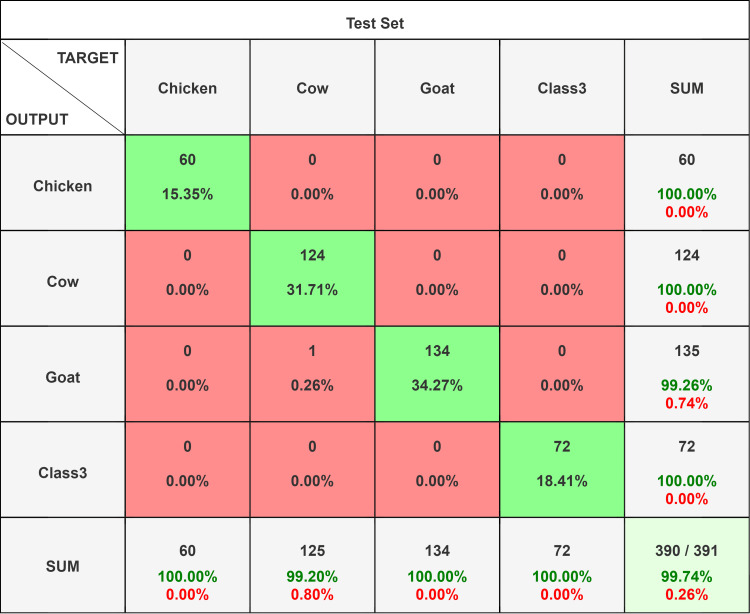
Confusion matrix comparison of different network models on test set ResNet 34. A total of 391 (72 human, 124 cow, 135 goat, and 60 chicken blood film images) original images were used for testing

**Figure 4:**
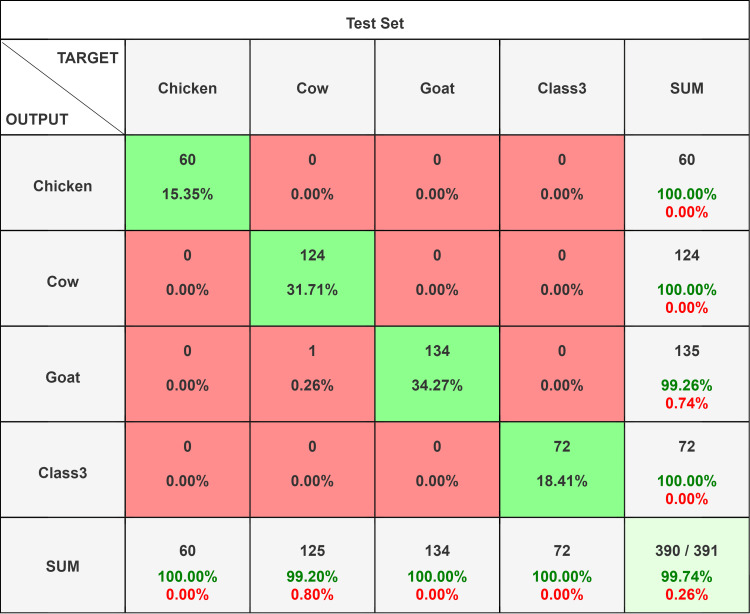
Confusion matrix comparison of different network models on the VGG16 test set. A total of 391 original images (72 human, 124 cow, 135 goat, and 60 chicken blood film images) were used for testing.

The overall framework of this research is based on four steps, namely, data accusation, processing, classification, and evaluation process. The test set was used to evaluate the model performance of the four network structures, namely, VGG16, ResNet18, and ResNet34 algorithms. Based on the image analysis, confusion matrices were generated (Figures 2-4), and the sensitivity, specificity, positive predictive value, negative predictive value, F1 scores, and MCC were calculated.

According to the findings, the F1 scores for the chicken, cow, goat, and human classes were all equal to 1.0 for each of the three algorithms. The MCC was 1 for chicken, cow, and human classes in all three algorithms, while the MCC score was 0.989 for the goat class by ResNet18, and it was 0.994 for both the ResNet34 and VGG16 algorithms. The results are shown in Table 2.

**Table 2:** Comparison of the results of three different algorithms for the accurate diagnosis of red blood cells of different four species

Species		ResNet18	ResNet34	VGG16
Chicken	Sensitivity	100%	100%	100%
	Specificity	100%	100%	100%
	Positive predictive value	100%	100%	100%
	Negative predictive value	100%	100%	100%
	F1 score	1	1	1
	Matthews correlation coefficient	1	1	1
Cows	Sensitivity	100%	100%	100%
	Specificity	100%	100%	100%
	Positive predictive value	100%	100%	100%
	Negative predictive value	100%	100%	100%
	F1 score	1	1	1
	Matthews correlation coefficient	1	1	1
Goat	Sensitivity	100%	100%	100%
	Specificity	99.2%	99.6%	99.6%
	Positive predictive value	98.5%	99.3%	99.3%
	Negative predictive value	100%	100%	100%
	F1 score	1	1	1
	Matthews correlation coefficient	0.989	0.994	0.994
Human	Sensitivity	100	100	100
	Specificity	100	100	100
	Positive predictive value	100	100	100
	Negative predictive value	100	100	100
	Precision	1	1	1
	Recall	1	1	1
	F1 score	1	1	1
	Matthews correlation coefficient	1	1	1

## Discussion

Deep machine learning techniques are employed to develop a computer vision-based system to analyze the microscopic, digitized images of various human and non-human tissues via different types of deep neural networks. To the best of our knowledge, this is the first time that deep machine learning has been used as a differentiating tool to recognize the origin of different blood stains detected in a crime scene. The findings of the present study revealed that the application of ResNet18, ResNet34, and VGG16 algorithms for the detection of RBCs of chickens, cows, goats, and humans yielded excellent results. These deep learning algorithms can effectively classify RBC cell images based on the morphological features of these cells. The higher scores of F1 and MCC of these ResNet18, ResNet34, and VGG16 algorithms revealed that the computer vision-based algorithms could be very helpful to pathologists for the differentiation of blood samples of the different species.

The recent advances in digital technology have revealed astonishing outcomes in many fields, including human health care. Digital technology has not only increased task performance speed but also improved accuracy with cost-effectiveness. It has also reduced the risk of errors, which could result in devastating consequences in terms of human health. There is a significant contribution of artificial intelligence in yielding fruitful results in digital technology. Artificial technology improves computational technology so that the computer can perform a task in a way similar to humans or even better. In the artificial intelligence technique, computer systems are made to learn and improve on their own from available data. Artificial intelligence has been used for speech recognition, analysis of radiological images, and evaluation of histopathological images [13, 17-20].

In the present study, the application of deep machine learning technology revealed very encouraging results and differentiated among the RBCs of humans, cows, goats, and chickens with very high reliability. The RBCs of these species exhibit variations in shape and diameter, which could be the basis of differentiation. The diameter of a human RBC is 7.5-8.7 μm [7]. The average diameter of a cattle RBC is 5.12 μm, and that of a goat is 3.39 μm [8]. A chicken RBC is oblong-shaped with an average length of 11.93 μm and a diameter of 7.13 um [21]. This measurement of the RBC diameter and the evaluation of the shape of these cells require special techniques and experience in this field. There is a dearth of experts in the field of forensic medicine. In this regard, the application of artificial technology may be quite useful as an aid in this matter. To the best of our knowledge, the current study was the first to use deep machine learning in this content. Additional studies using different algorithms will further pave the way for the application of AI technology in this field. Moreover, the technique needs to be rechecked for the differentiation of blood stains after different time points to check its ability to differentiate the origin of blood stains with less fresh and old stains with distortion of the normal shape of RBCs.

## Conclusions

The application of artificial intelligence with deep machine learning is widely investigated in the medical field in the identification of histopathological specimens of different diseases. This work was conducted to assess the application of the technique in the identification of the origin of RBCs in fresh blood stains that can be collected from crime scenes. This work proved that the computer vision-based system developed by artificial intelligence showed excellent performance in the differentiation of human RBCs from other species (chickens, cows, and goats) blood cells, which could be a very useful adjunct tool in the process of investigation of various crime scenes.
